# Prevalence of oral complications in the course of severe SARS-CoV-2 infection under mechanical non-invasive ventilation

**DOI:** 10.1186/s40001-023-01273-6

**Published:** 2023-08-22

**Authors:** Elzbieta Paszynska, Maria Gawriolek, Amadeusz Hernik, Justyna Otulakowska-Skrzynska, Hanna Winiarska, Daria Springer, Magdalena Roszak, Zuzanna Slebioda, Anna Krahel, Szczepan Cofta

**Affiliations:** 1 Department of Integrated Dentistry, Poznan University of Medical Sciences (PUMS), Bukowska st. 70, 60-812 Poznan, Poland; 2 Department of Pulmonology, Allergology and Respiratory Oncology, Poznan University of Medical Sciences (PUMS), Szamarzewskiego 82/84, 60-569 Poznan, Poland; 3 Department of Computer Science and Statistics, Poznan University of Medical Sciences (PUMS), Rokietnicka st. 7, 60-806 Poznan, Poland; 4 Department of Gerodontology and Oral Pathology, Poznan University of Medical Sciences, 60-812 Poznan, Bukowska st. 70, Poland

**Keywords:** Covid-19, Hospital treatment, Oral health, Pneumonia

## Abstract

**Background:**

The management of oral health during severe symptoms of Covid-19 is still a challenge, especially in intensive care units under invasive/noninvasive ventilation in hospital. Understanding the cause-and-effect relationships may allow for individual adjustment of oral care recommendations during Covid-19 disease. The study’s objective was to assess Covid-19 patients’ oral health status under hospital treatment due to pulmonary adverse Covid-19 outcomes.

**Material and methods:**

Covid-19 patients (mean age 74.4 ± 15.4; *n* = 120, male *n* = 50/female *n* = 70) were admitted to hospital in the acute phase of Covid-19 between January and March 2022 who required oxygen therapy due to pneumonia, rapid respiratory failure, low saturation. Blood and radiological tests were taken according to National Health Fund guidelines. The condition of teeth (Decayed, Missing, Filled teeth as DMFT index), dental hygiene (Plaque Control Record as PCR index), periodontal status (probing depth PD, clinical attachment CAL, bleeding on probing BOP) and oral mucosa (BRUSHED and Beck scores) were examined.

**Results:**

Charateristics of the teeth (dental caries 35.2%, DMFT Median 22), plaque retention (83.4%), advanced periodontitis (48.3%), xerostomia (74.2%), oral mucosa inflammation (80.8%), angular cheilitis (53.3%), hemorrhagic (21.7%) showed a high incidence of harmful oral conditions. BRUSHED model and Beck score indicated moderate oral dysfunction and need for oral care every 8 h. Spearman’s analysis revealed a significant positive correlation between pneumonia and neutrophile, interleukin-6 IL-6, C-reactive protein CRP (*p* = 0.01, *p* < 0.001, *p* < 0.001), negative to lymphocyte count (*p* < 0.001). Multiple and logistic regressions selected the following risk predictors for pneumonia as IL-6, CRP, obesity and for severe COVID-19 symptoms D-dimer level and a lack of targeted vaccination (*p* < 0.001). Among oral predictors, the PCR index and Beck score were significant for both outcomes (respectively *p* < 0.001, *p* < 0.012). Patients who received oxygen therapy with face masks had more often angular heilitis and debris (*p* = 0.025, *p* = 0.035).

**Conclusions:**

COVID-19 hospitalised patients with severe symptoms crossing with poor oral health-related conditions. This may exacerbate a response for COVID infection, and play a role in cytokine storm. For Covid-19 management, to inhibit extraoral/intraoral complications, it is recommended to adjust oral hygiene procedures, including antibacterial, protective, moisturising agents after individual oral health assessment.

**Supplementary Information:**

The online version contains supplementary material available at 10.1186/s40001-023-01273-6.

## Introduction

Patients hospitalised due to SARS-CoV-2 infection causing pathological changes in the oral cavity are still a novel challenge for dental care [1, 2]. A wide range of medical symptoms has been observed in the course of Covid-19 [3, 4]. The most common are: fever, cough, shortness of breath or difficulty breathing, fatigue, and muscle pain [3, 5]. New reports continue to emerge on the symptoms and complications of SARS-CoV-2 infection—including those located in the oral cavity [6–8]. Therefore, oral evaluation accompanying Covid-19 disease in patients undergoing intensive treatment may support medical care.

Update studies have confirmed that ACE2 receptors of the SARS-CoV-2 virus infecting human cells are detected not only in the upper respiratory tract but also in the epithelial cells of the oral mucosa, salivary ducts, and tongue [9, 10]. Probably good oral hygiene may reduce the oral cavity’s viral load and prevent infection from the oral cavity to the lower respiratory tract [11–13]. Unfortunately, questionnaire studies imply that the COVID-19 pandemic was a critical transition period for increasing risk in decreasing hygienic behaviours [5, 14, 15]. Poor tooth brushing and plaque accumulation induce not only periodontal diseases and dental caries but the dissemination of bacteria into the bloodstream, inflammatory activity and potential risk for systemic diseases [16–22]. Previous studies suggest that oral decontamination reduces the likelihood of ventilator-associated pneumonia (VAP) and may reduce other nosocomial infections [11, 12, 20].

The present study’s objective was to assess Covid-19 patients’ oral health status under hospital treatment due to pulmonary adverse Covid-19 outcomes. The null hypothesis was that there would be no significant signs of oral disease.

## Material and methods

University Review Board approval (No. 92/22) was obtained. Subjects were recruited among the patients admitted to the single State-run hospital designed for severe cases of Covid-19. The clinical trial was conducted in accordance with the ethical standards outlined in the 1964 Declaration of Helsinki and its subsequent amendments, and in accordance with the Good Clinical Practice guidelines of the International Conference on Harmonization (ICH). Before the dental examination, a medical history was completed and read for each subject, and an informed consent form was signed.

### Material

A temporary reference hospital (public) was organized for intensive care of Covid-19 patients from October 2021 to March 2022. The examined patients were admitted in the acute phase of Covid-19 to be treated from January 2022 to March 2022. The study group consisted of one-hundred-twenty patients (*n* = 120, male *n* = 50/ female *n* = 70); consecutive patients aged from 22 to 95 years old were transferred from the mid-west part of the country. After testing positive for Covid-19, all patients continued regular tests during hospitalisation. Inclusion and exclusion criteria are presented in Table 1.

**Table 1:** Inclusion and exclusion criteria for the study group

Criteria for inclusion into the study group	Criteria for exclusion from the study group
Male/female patients aged > 18 y	Male/female patients aged < 18 y
Patients diagnosed with Covid-19 under ICD-10 diagnostic criteria (diagnosis confirmed by reverse-transcription polymerase chain reaction test (qRT-PCR)	Patients with negative Covid-19 test (qRT-PCR)
Severe status of Covid-19 symptoms [23, 24]	No Covid-19 symptoms at present, sepsis or multiresistant bacterial infection
A patient approval for intraoral/periodontal examination	Lack of acceptance from patients for intraoral/periodontal examination
Hospital admission and full data availability from medical records	Missing data regarding present health status, discharge, death
Non-invasive ventilation treatment	Invasive ventilation support: mechanical or tracheostomy ventilators
Performance of intraoral/periodontal examination No orthodontic treatment	Difficulties in intraoral/periodontal examination Orthodontic treatment
No radiotherapy for the head/neck area	Receiving radiotherapy for the head/neck area

Data analysis included also comorbities that are well-known risk factors for increase the risk of severe COVID-19 course of the disesase and mortality. Data were retrieved from medical charts registered at hospital admission based on objective measurements, medical history and self-reported data. At the beginning of hospitalisation, every patient has blood tests: complete blood count, asparagine transferase (AST), alanine transferase (ALT), gamma glutamyl-transpeptidase (GGTP), lactate dehydrogenase (LDH), C-reactive protein (CRP), procalcitonin (PCT), IL-6 interleukin-6 (IL-6), creatinin, D-dimer, activated partial thromboplastin time (APTT), international normalised ratio (INR), glucose level, lipid profile, natrium (Na^+^), kalium (K^+^), protein electrophoresis, hepatitis B surface antigen (Hbs ag), anti-human immunodeficiency virus antibodies (anti-HIV), anti-hepatitis C antibodies (anti-HCV)—according to National Health Fund guidelines. All the patients had a radiological test: mostly high-resolution computed tomography (HRCT). The chest X-ray was performed only on two groups of patients: without any clinical symptoms of pneumonia or in the group of patients with highly severe respiratory failure who could not survive the HRCT procedure. The study dependent data included COVID-19 outcomes achieved at the end or during hospitalization, follow WHO criteria [23, 24]:

1. severity of symptoms, classified as mild, moderate, severe or critical
2. hospital admission and discharge criteria, numbers of days in hospital, admission to Intensive Care Unit (ICU)
3. hospitalization endpoint, discharge or death
4. type of ventilation needed, as mechanical ventilator (invasive and noninvasive)
5. clinical manifestations and symptoms

### Oral examination

The clinical evaluation was carried out under a complete sanitary regime using disposable, sterile diagnostic kits under artificial illumination and forehead light. The clinical oral examination included elements such as oral hygiene, periodontal status, evaluation of dental caries incidences, and general oral conditions by the BRUSHED model and Beck’s score (see description below) [25–31]. Before the study, two qualified dentists were trained and calibrated according to generalized periodontitis criteria (stage III), for non-Covid-19 patients (EP, MG). Oral examiners worked weekly in three-person dental teams (examiner and two dental assistants ZS, AH, AK, JO-S) under sanitary protocol prepared for the medical personnel of a Covid-19 unit. Inter- and intra-examiner reliability was acceptable for the oral examination parameters because the ICC values and Cohen's Kappa coefficient were ≥ 0.8 (*p* < 0.001).

Dental plaque condition was recorded using a manually graded periodontal WHO probe (LM-instruments, LM8 5050 probe, Osakeyhtiö, Parainen, Finland). The instrument consisted of a 0.5 mm ball at the tip, with mm markings at 3.5, 8.5, and 11.5 mm and colour-coding from 3.5 to 5.5 mm. The probing was performed using only gentle probing forces with a periodontal probe of appropriate size (force 0.25–0.30 N). Plaque control was evaluated using the dichotomised Plaque Control Record index (PCR). The proportion of surfaces (%) with dental plaque was calculated as % of sites [26–28].

Periodontal status was categorized as healthy, gingival inflammation or periodontitis, per the classification of periodontal disorders and clinical manifestations [29]. Periodontal measurements were based on the following parameters: probing depth (PD), clinical attachment (CAL), and bleeding on probing (BOP). Periodontal status was evaluated in six locations per tooth in all teeth (among dentate patients). Gingival health was assessed, resulting from < 10% bleeding sites with PD ≤ 3 mm. Gingival inflammation was graded as ≥ 10% bleeding sites with PD ≤ 3 mm, periodontitis was defined as a confirmed interdental CAL ≥ 2 mm non-adjacent teeth, or buccal or lingual CAL ≥ 3 mm with pocketing ≥ 3 mm at ≥ two teeth (not attributed to causes unrelated to periodontitis) [29]. No X-ray pictures for any dental/periodontal status were taken due to the temporary status of the units and the restricted area of the Covid-19 zone.

After cleaning and drying (excluding the third molars), the teeth surfaces were scored under good dental lighting, without magnification [25]. Dental examination records included the number of carious teeth, the number of restored teeth by fillings, and the number of missing teeth due to caries, using the Decayed, Missing, Filled teeth (DMFT) index evaluating dental caries [25].

The general condition of the oral cavity and the condition of the oral mucosa were assessed in all patients using a standard dental kit under artificial light. The type and the location of pathologic lesions were documented. Oral assessment of soft tissues was performed using the BRUSHED model, established by Hayes and Jones [16, 30] and presented in Additional file 1: Table S1. The presence of bleeding, redness, ulceration, saliva, external factors and debris were noted for each patient. Due to wearing of face masks by all patients and dental teams in the infected zone of the hospital—the presence of halitosis was excluded from oral examination.

Beck's oral assessment tool. To achieve individual indications for oromucosal care, Beck’s oral assessment model was used [31]. This tool was initially developed for assessment of stomatitis post chemotherapy and adopted with modification for intensive care units. Patients were evaluated in five areas: lips, gingival and oral mucosa, tongue, teeth, and saliva. In this assessment system, grades from 1 to 4 points may be assigned in each category depending on the degree of clinical symptoms. A total score indicates the current level of oral mucosa dysfunction (from none to severe) and suggests a suitable oral care protocol. Beck's score is presented in Additional file 2: Table S2 in the detailed description.

### Data analysis and statistics

The analyzed data were expressed as mean ± standard deviation, median, minimum and maximum values or percentage, as appropriate. Normality of distribution was tested using the Shapiro–Wilk test and the equality of variances was checked with Levene’s test. The relationship between variables was analyzed with Spearman’s rank correlation coefficient and by multiple regression (e.g., involvement of the lung parenchyma with pneumonia). Categorical data were analyzed with the *χ*^2^ test or the Fisher-Freeman-Halton test. Statistical analyses were performed with STATISTICA 13.0 (StatSoft Inc., Tulsa, USA) or StatXact 11.0 (Cytel Inc., Waltham, Massachusetts, USA). In addition, to determine risk factors for those significantly affecting the occurrence of death or symptoms (dichotomous dependent variables), a logistic regression was also carried out, odds ratio and 95% confidence intervals were set for the indicated variables. The independent variables were entered in the model in a forward block-wise design that included vaccination against Covid-19, comorbidities (diabetes, hypertension, cardiovascular diseases, obesity) and biochemical parameters (D-dimer, C-reactive protein, interleukine IL-6, lymphocyte levels) and oral health-related predictor variables (DMFT index, number of carious teeth, PCR index, periodontitis and Beck’s score). The patient’s age was included as a potential confounder in all multivariate models. Logistic regression calculations were performed in a statistical package MedCalc v. 19.5.1 (MedCalc Software, Ostend, Belgium). All results were considered significant at *p* < 0.05.

## Results

**Fig. 1:**
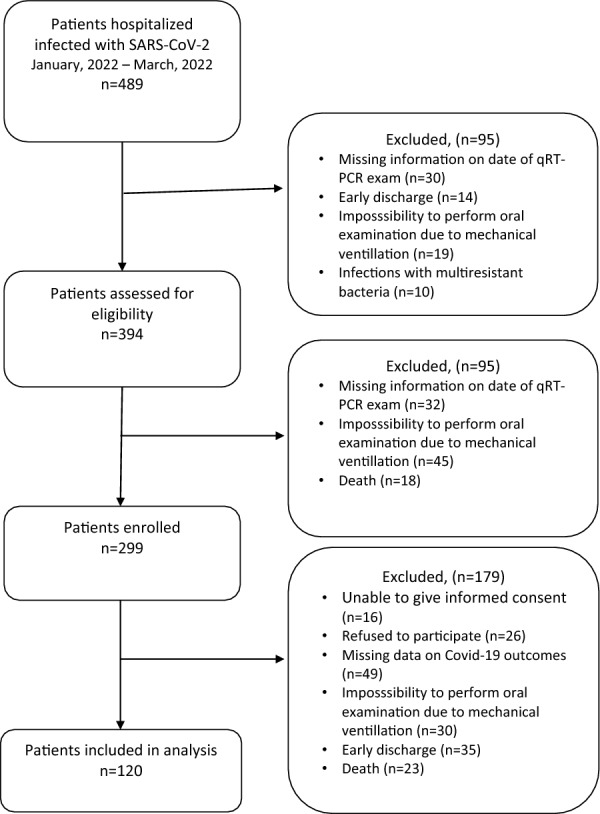
Supporting information on flow chart of the study participants

During the nearly 3-month recruitment and data collection period, research teams visited the hospital 12 times (on average, once a week). A total of 394 hospitalised patients were eligible to participate in the study during visits. However, 75 patients had not been available for a dental examination before the hospitalisation endpoint due to mechanical ventilation and were excluded or missing information on date of qRT-PCR Covid-19 tests (*n* = 81). Other 299 patients were assessed for eligibility, but 16 of them were unable to give informed consent, refused to participate (*n* = 26), died (*n* = 23), missing data on Covid-19 outcomes (*n* = 49) or were discharged early (*n* = 35). Finally, 120 patients were assessed from total baseline and follow-up data and were included in the analysis (supporting Flowchart is available in online as Fig. 1).

The main characteristics of the participants are detailed in Table 2. The age ranged from 22 to 95 years (mean = 74.4 ± 15.4), 47.1% were *M*, and the patients came from within the Poznan agglomeration in the country’s mid-Western region. There was a high incidence of comorbidities (94.2%). Table 2 shows the incidence of these diseases and the characteristics of hospitalisation regarding the total length of hospital stay, such as general health vaccination status, against Covid-19, length of hospitalisation, the severity of symptoms and hospital endpoint. Most hospitalised patients were observed to have had one or more coexisting symptoms.

**Table 2:** Baseline, general symptoms data and final hospital endpoint of patients hospitalized in Covid-19 state-run hospital due to Covid-19 (*n* = 120)

Variables	Categories	*n *(%)
Sociodemographic		
Sex	Male	50 (41.7)
	Female	70 (58.3)
Age^a^ 74.4 ± 15.4	≤ 30 y	4 (3.3)
	> 30 to 50 y	7 (5.8)
	> 50 to 65 y	12 (10.0)
	≥ 65 y	97 (80.9)
Race	White	120 (100.0)
BMI^a^ 28.03 ± 4.9	score < 30	88 (73.3)
	score ≥ 30	32 (26.7)
Hospital	Covid-19 state-run hospital	120 (100.0)
Time of the oral examination after admission (in days)	≤ 7 days	23 (19.2)
	7–14 days	66 (55)
	≥ 15 days	31 (25.8)
General health status	Diabetes	22 (18.3)
	Hypertension	68 (56.7)
	COPD	32 (26.7)
	Cardiovascular diseases	61 (50.8)
	Alcohol intake (present and former)	9 (7.5)
	Smoking (present and former)	5 (4.2)
	Dementia	22 (18.3)
	Cancer	25 (19.2)
	Neurological diseases	21 (17.5)
	Obesity	32 (26.7)
	Arthritis	26 (21.7)
	Osteoporosis	17 (14.2)
	Thyroid disease	12 (10.0)
Three-dose Covid-19 vaccination	Yes	63 (52.5)
	No	57 (47.5)
Level of symptoms	Critical	5 (4.2)
	Severe	10 (8.3)
	Moderate	55 (45.8)
	Mild	50 (41.7)
Hospital endpoint	Discharge	66 (55.0)
	Death	23 (19.2)
	Discharge to another care facility	29 (24.2)
	Self-discharge	2 (1.7)
	Number of days in hospital^a^	13.0 ± 9.1
Laboratory parameters^b^	Neutrophile	76.3 (9.8–825)
	WBC	6.0 (1.4–36.4)
	Lymphocyte	14.5 (1–79.9)
	Platelets	217.5 (67–569)
	CRP	40 (4–1278)
	IL-6	14.1 (0.1–2402)
	D-dimer	1431 (190–23051)

COPD, chronic obstructive pulmonary disease; y, years; n, number of patients; Neutrophile (× 10^3^/μl); WBC, White Blood Cell (× 10^3^/μl); Lymphocyte (× 10^3^/µl); Platelets, cell/mL × 10^3^/μl; CRP, C-reactive protein (mg/l); IL-6, Interleukin-6 (pg/ml); DD, D-dimer (µg/l). Number of days in hospital as Median (Min–Max)—10 (3–57)

^a^Mean ± standard deviation

^b^Median (Min–Max)

Patients required hospital treatment due to severe course of Covid-19, such as pneumonia, acute respiratory failure, blood saturation decrease and (very few patients) Covid-19-positive persons who required hospitalisation because of other comorbidities.

Symptom status ranged from mild in fifty patients (41.7%) to seriously critical in 5 cases (4.2%). Twenty-three patients (19.2%) died during hospitalisation. The length of hospital stay ranged from 3 to 57 days. Sixty-six (55%) patients were discharged home due to remission of symptoms and Covid-19 infection after hospitalisation. Twenty-nine (24.2%) were discharged to another facility, and 2 (1.7%) were discharged spontaneously from the hospital.

The condition of teeth, periodontium and oral mucosa, are presented in Tables 3 and 4. Overall characteristics showed a high incidence of harmful oral condition. Nearly fifty-three patients (44.2%) were completely edentulous, and fifty-seven (47.5%) used partial dentures. In the group of patients with dentition (*n* = 67), the frequency of dental caries was 35.2%, and the dental treatment index was calculated at 0.4. The median DMFT score was 22, and the plaque retention by PCR% was 83.4 ± 21.6. Advanced periodontitis was diagnosed among 58 (48.3%) participants with teeth or partial teeth. Concerning the BRUSHED model, xerostomia was found in 89 patients (74.2%), oral atrophy and inflammation in 97 (80.8%), angular cheilitis in 64 (53.3%), visible plaque and external particles in 100 (83.3%) and vascular and hemorrhagic changes in 26 (21.7%). Beck’s oral assessment median at 14 was estimated as moderate oral dysfunction with an indication for oral care every 8 h.

**Table 3:** Oral status of hospitalized COVID-19 patients (*n* = 120)

Variables	Category/parameter	Mean ± standard deviation
Number of teeth^a^ (*n* = 67)	Present	9.2 ± 11.1
	Decayed	2.1 ± 4.7
	Missing	22.8 ± 11.1
	Filled	1.4 ± 3.3
PCR (% of sites)^a^ (*n* = 67)	–	81.3 ± 17.8

		*n* (%)
DMFT index^a^ (*n* = 67) 22.01 ± 8.7	≤ 6	3 (5)
	7–14	14 (11.7)
	15–21	16 (13.3)
	≥ 22	87 (72.5)
Fully edentulous	–	53 (44.2)
Use of dentures	No	67 (55.8)
	Yes—maxillary	11 (9.2)
	Yes—mandibular	2 (1.2)
	Yes—both	44 (36.7)
Periodontal status *n* = 67	Healthy	0 (0.0)
	Gingivitis	9 (13.4)
	Periodontitis	58 (48.3)
Highest probing depth *n* = 67	≤ 3 mm	9 (13.4)
	≥ 4 and ≤ 6	52 (43.3)
	≥ 7 mm	6 (9.0)
Beck’s score^a^ *n* = 120 13.6 ± 4.1	0–5 points	7 (5.8)
	6–10 points	19 (15.8)
	11–15 points	60 (50.0)
	16–20 points	34 (28.3)

*n* number of patients, *DMFT* Decayed, Missing, and Filled Teeth index, *PCR* Plaque Control Record index, *Beck score* Oral Assessment Tool

^a^Mean ± standard deviation

**Table 4:** Results of BRUSHED assessment model (*n* = 120)

Symbolic letter	Category/parameter	*n* (%)
B	BLEEDING (gums, mucosa, coagulation status)	26 (21.7)
R	REDNESS (gum margins, tongue, stomatitis)	97 (80.8)
U	ULCERATION	5 (4.2)
S	SALIVA characteristic xerostomia	89 (74.2)
H	HALITOSIS^a^	xcluded from clinical oral examination
E	EXTERNAL FACTORS angular heilitis	64 (53.3)
D	DEBRIS (visible plaque, external particles)	100 (83.3)

^a^Excluded from oral examination due to non-invasive oxygen ventilation by mouth

### Correlations

This data set is summarized in Table 5. Spearman’s analysis of the Covid-19 group results revealed a clear correlation between D-dimer level and DMFT index, missing, filling teeth, highest probing depth (*p* = 0.004, *p* < 0.001, *p* = 0.02, *p* < 0.001, *p* = 0.018). A similar correlation between Beck score and D-dimer, lymphocyte, WBC, neutrophile, IL-6 levels (respectively *p* = 0.018, *p* = 0.02, *p* = 0.019, *p* = 0.003, *p* = 0.001) was also evidenced. According to BRUSHED score there were positive correlation between patients who received oxygen therapy by face masks and angular heilitis or debris (*p* = 0.025, *p* = 0.035).

**Table 5:** Significant results of the Spearman’s/Pearson’s correlation rank tests regarding clinical and biochemical parameters (*p* < 0.05) for the Covid-19 patients

Correlations between variables *n* = 120	*p*-value	Spearman *R* /Pearson *r*	Correlations between variables *n* = 120	*p*-value	Spearman *R* /Pearson *r*
DD & DMFT index	**0.0001**	0.35	Neut & Beck score	**0.003**	0.27
DD & M	**0.0002**	0.34	Neut & saturation	**0.011**	− 0.23
DD & F	**0.002**	− 0.29	Neut & pneumonia	**0.001**	0.29
DD & number of teeth	**0.0002**	− 0.33	Lymphocyte & pneumonia	**0.00001**	− 0.47
DD & highest probing depth	**0.0002**	0.33	IL-6 & Beck score	**0.001**	0.29
DD & Beck score	**0.018**	0.22	IL-6 & number of days in hospital	**0.017**	0.21
DD & age	**0.01**	0.23	IL-6 & saturation	**0.001**	− 0.29
DD & number of days in hospital	0.081	0.18	IL-6 & pneumonia	**0.0004**	0.32
DD & como rbidity	**0.025**	0.20	CRP & PCR	0.056	− 0.18
Lymphocyte & Beck score	**0.002**	− 0.28	CRP & number of days in hospital	**0.005**	0.26
Lymphocyte & saturation	**0.013**	0.23	CRP & saturation	**0.003**	− 0.27
WBC & Beck score	**0.019**	0.21	CRP & pneumonia	**0.000001**	0.44
			CRP & comorbidity	**0.028**	0.21

DD, D-dimer (µg/l); WBC, White blood cell (× 10^3^/µl); CRP, C-reactive protein (mg/l); IL-6, Interleukin-6 (pg/ml); Lymphocyte count (× 10^3^/µl); Neut, Neutrophile count (× 10^3^/µl); PCR, Plaque Record (% of sites); *D*, number of decayed teeth; *M*, number of missing teeth due to caries; F, number of filled teeth due to caries; DMFT index, decayed, missing and filled teeth. Results of significant correlation are expressed in bold *p-value*.

Analysis of the general health and biochemical data according to pneumonia revealed a significant correlation with neutrophile, IL-6, CRP levels (*p* = 0.01, *p* < 0.001, *p* < 0.001), as was found negative between pneumonia and lymphocytes (*p* < 0.001). Saturation was correlated with lymphocytes (*p* = 0.013) and negatively with neutrophile, IL-6, CRP (*p* = 0.011, *p* = 0.001, *p* = 0.003). Correlations were also observed between mentioned above variables in Table 5.

To explain which of the risk factors had an impact on pneumonia severity, a multiple regression model was set with 10 variables in Block 1, of which seven predictors were obtained (*p* < 0.001), of which three occured significant: IL-6, CRP, obesity (*p* < 0.05) and two variables were close to significance: D-dimer and lymphocyte levels (*p* = 0.074, *p* = 0.073). For Block 2, there were two significant variables with the impact on both, pneumonia and severity of COVID-19 symtoms as PCR index (respectively, *p* = 0.007, *p* = 0.028) and Beck score (respectively, *p* = 0.005, *p* = 0.11). Data are presented in Table 6.

**Table 6:** Risk estimation of the association between COVID-19 outcomes and selected independent variables

	b*	*p*-value
Block 1 *n* = 120		
Multiple regression forward, dependent variable: pneumonia, *R* = 0.55, *R*^2^ = 0.30, *p* < 0.001		
Intercept	− 1117.99	0.019
IL-6	0.38	**0.00002**
CRP	0.24	**0.005**
Obesity	0.20	**0.018**
DD	0.15	0.074
Lymphocyte	0.15	0.073
Sex male/female	ns	0.159
Vaccination	ns	0.279
Block 2 *n* = 67/120		
Multiple regression forward, dependent variable: pneumonia, *R* = 0.52, *R*^2^ = 0.27, *p* < 0.001		
Intercept	13.21	0.467
PCR	− 0.36	**0.007**
Beck score	0.36	**0.005**
* D*	− 0.23	0.071
Sex male/female	ns	0.145
Periodontitis	ns	0.219
DMFT index	ns	0.125
Age	ns	0.115
Multiple regression forward, dependent variable: severe COVID-19 symptoms, *R* = 0.40, *R*^2^ = 0.16, *p* < 0.012		
Intercept	1.03	0.039
PCR	− 0.29	**0.028**
Beck score	0.32	**0.011**
Periodontitis	ns	0.151

Data are expressed as statistical significance are highlighted in bold

DD, D-dimer (µg/l); CRP, C-reactive protein (mg/l); IL-6, Interleukin-6 (pg/ml); Lymphocyte count (× 10^3^/µl); PCR, Plaque Control Record index (% of sites); *D*, number of decayed teeth; DMFT index, decayed, missing and filled teeth. In Block 2, for the following oral variables PCR, *D* and Periodontitis *n* = 67, for the rest oral variables *n* = 120

b*, regression beta coefficient

There were failures to estimate the model for Block 1 to account the risk of death. In the logistic regression only age turned out to be significant (*p* < 0.05).

A logistic regression was also carried out to determine risk factors significantly influencing degradation from mild to critical symptoms. Therefore, the connection between the probability of symptoms and the group of independent variables was considered. Parameters taken for analysis in accordance with previous studies and observations in the COVID-19 literature (Block 1) determined the model (*p* < 0.001) indicating three significant variables: vaccination (yes/not), D-dimer, CRP levels (*p* < 0.05). The both, D-dimer (OR 0.99), and COVID-19 vaccination (OR 0.38), determined the chance of severe symptoms decrease. The results of CRP (OR 1.1) indicated that an increase of one unit of CRP results in a 1.1 times greater chance of developing severe symptoms. The logistic regression in the oral Block 2 revealed only Beck score (OR 1.34) and age of patients (OR 1.07) as significant. Data are presented in Table 7.

**Table 7:** Risk estimation of the association between severe COVID-19 symptoms and selected independent variables

	Logistic regression backward, dependent variable: symptoms severity; Block 1 (*R* = 0.55, *R*^2^ = 0.31, *p* < 0.0001) and Block 2 (*R* = 0.57, *R*^2^ = 0.33, *p* < 0.0004)			
	Odd ratio	95% CI	Coefficient	*p*-value
Block 1 *n* = 120				
DD	0.99	0.99 to 1.0	− 0.0001	**0.026**
CRP	1.01	1.01 to 1.02	0.01	**0.0002**
Vaccination	0.38	0.16 to 0.90	− 0.97	**0.027**
Age	ns	ns	0.02	0.089
Block 2 *n* = 120				
Beck score	1.34	1.07 to 1.67	0.29	**0.011**
Age	1.07	1.01 to 1.13	0.06	**0.029**

Data are expressed as statistical significance are highlighted in bold

*DD* D-dimer (µg/L), *CRP* C-reactive protein (mg/l)

In the whole group of patients the oral findings such as periodontitis, number of decayed teeth D, score DMFT and dental plaque deposits PCR were not associated (*p* = 0.530, *p* = 0.878; *p* = 0.252; *p* = 0.447). However, the separation of obese patients with BMI > 30 showed trend similar to significance (*p* = 0.052). In addition, we compared IL-6 levels between patients with and without oxygen therapy. These subgroups differed (*p* = 0.006) and IL-6 levels were higher in patients with oxygen therapy.

## Discussion

The clinical study found the coexisting lesions in the hard and soft tissues of the oral cavity in patients hospitalised due to severe SARS-CoV-2 infection and the acute course of Covid-19. The vast majority of the respondents required intervention in the form of conservative/periodontal/surgical treatment. The negative impact of the Covid-19 disease on oral health has been demonstrated, manifested by increased exposure to poor hygiene, general dysfunction of the oral cavity combined with poor mucous membrane, and tongue and lips condition. Exacerbation of symptoms in the oral cavity was particularly significant in patients who were severe ill and required hospitalisation.

Oral health in patients with COVID-19 is often impaired due to several direct and indirect mechanisms. Therefore, not only the pathological nature of the virus itself should be considered. Coronavirus respiratory track invasion via the oral cavity enhances several immunologic reactions; cytokine storm caused by dysregulated humoral and cellular mechanisms can aggravate existing autoimmune conditions affecting the oral mucosa [32]. Comorbidities, intensive pharmacotherapy, oxygen therapy and older age of patients were also significant [33, 34]. These variables assessed in this study may be confounding and contribute to poorer oral health. So far, no data are available on the oral cavity condition in Polish patients hospitalised during the acute course of Covid-19. From the other hand, international data were based on various research algorithms and are not uniform, which makes it difficult to compare the results.

In present study, periodontitis occured significant frequently in the severe effects of COVID-19 disease. Comparing national epidemiological studies in persons aged 65–74, healthy periodontitis has on average 5%, gingivitis occurs in 33%, and periodontitis in 14% [35, 36]. Our results are higher than the national ones, probably due to the older age of patients, numerous comorbidities, pharmacotherapy and reduced immunity due to the need for hospitalisation and the severe course of Covid-19. Similar results of periodontitis in patients hospitalised by Covid-19 were obtained [10, 37–39]. Lloyd-Jones [40] proposed a hypothesis about the potential role of periodontitis. Systemic inflammatory processes seen in severe Covid-19 patients and periodontitis may exacerbate SARS-CoV-2 infection [7, 8, 41, 42]. Although our results cannot be considered direct etiological evidence, they do provide solid evidence of a periodontitis-Covid-19 relationship, even after accounting for significant comorbidities. One of the recent molecular tests confirmed that SARS-CoV-2 could also be detected in periodontal pockets and caries lesions which may serve as reservoirs for the virus. However, the sensitivity of SARS-CoV-2 detection is low compared with other methods [10]. With regard to the state of partial or complete edentulism, nearly 50% of patients with Covid-19 used some prosthesis, and a similar number were classified as edentulous. Recent national studies from various regions indicate that the percentage of edentulousness is from 13 to 59% of the elderly population [35, 43]. Local factors associated with tooth loss also predispose individuals to severe inflammatory and immune responses, especially when periodontal disease is the primary cause of tooth loss [44]. The viral disease could impact on shift in the oral microbiome [45]. With regard to the high DMFT in our group of patients, it is highly probable that these patients were affected by untreated dental caries and periodontal disease before viral infection and hospitalisation [46]. National research in this age group shows that the incidence of caries may range from 30% in older adults living with their families to even 100% in residents of Social Care Centres [36]. Based on panoramic X-rays the records of Covid-19 patients, the relationship between the dental damage stage and the severity of Covid-19 was found to be remarkable. It should be emphasized that an individual approach is needed to understand the specificity of problems in the elderly group of patients. It also allows for setting priorities for a given patient. Caries and periodontal disease in the elderly have a chronic course without subjective pain symptoms [34, 47]. If left untreated, dental caries ultimately leads to pulpitis, tooth loss, and even severe systemic consequences [48]. Surveys conducted during the pandemic since 2022 show that there was a willingness among seniors to postpone dental visits [14, 15, 46].

In present study, patients with Covid-19 also had a high incidence of angular heilitis and debris accumulation with regard to BRUSHED score. Undoubtedly, this was influenced not only by a virus infection, impaired immunological deficiency, severe general condition of patients, face masks for oxygen supply making food intake difficult, but also isolation in a temporary hospital, lack of contact with family, complete dependence on hospital staff working in the sanitary regime. All these factors made it difficult to maintain oral hygiene. Presumably, food debris retention, mouth breathing, and desiccation of the oral mucosa in seriously ill patients have caused dysbiosis in the microbial community, including the colonisation of anaerobic Gram-negative strains [20, 49–52]. Studies before the pandemic outbreak confirmed that good oral hygiene measures might prevent the spread of infection from the oral cavity to the lower respiratory tract and pneumonia risk [53]. The incidence of pathological changes on the oral mucosa in the study group was very high, and what is interesting, many persons had different lesions coexisting. The most common abnormalities were: mucosal plaque, atrophic-inflammatory changes, dry mouth and angular cheilitis. Less frequently, vascular and hemorrhagic lesions were observed.

The presence of a removable coating on the surface of the oral mucosa in hospitalized patients may be a consequence of limited oral hygiene and a change in diet during illness (consumption of soft, mushy products). It may also indicate the development of a fungal infection favoured by immunological disorders. Some of the patients developed symptoms characteristic of geographic tongue, the signs of which were irregularly distributed coating and hypertrophy of the filiform papillae of the tongue. Exacerbations of autoimmune diseases are also described in the course of Covid-19 infection [2]. Plaque on the oral mucosa is also more common in persons with impaired salivation. Based on the BRUSHED model and the Beck scale, it was estimated that the vast majority of patients showed dryness of the oral mucosa, revealed almost in 75% of the examined subjects. This symptom can be induced directly by the virus and indirectly, as a consequence of drug therapy, stress and anxiety, or nutritional deficiencies that often accompany the infection. SARS-CoV-2 presents specific neurotropic and mucotropic abilities and may impair salivary glands’ functioning, taste and smell sensation and oral mucosa integrity [54]. It has been observed that SARS-CoV-2 binds angiotensin-converting enzyme 2 (ACE2)-positive cells, (ACE2) and transmembrane protease serine 2 (TMPRSS2)-positive cells, which makes the salivary glands a potential target for the virus [55–57]. SARS-CoV-2 infection may lead to salivary gland inflammation and damage via the immunopathological routes [2, 33]. Reduced saliva flow may secondarily result in several other oral complications, including an increased risk of opportunistic infections or trauma [1].

Among the indirect causes of the oral mucosa dryness in the examined group of patients, intensive oxygen administration, covering not only the nasal passages but the oral cavity (mouth breathing), decreased water intake, stimulation of masticatory mechanoreceptors, comorbidities and intensive drug therapy were of crucial importance. Patients under oxygen treatment maintain their mouths open and exacerbate dryness with potential oral infections [32, 58].

The mucous membranes of the examined patients, the atrophic and inflammatory changes of the oral mucosa, such as cracked lips, and inflammation of the lip and tongue, were noted. This clinical picture resembled the symptoms of Kawasaki disease [2]. This was in line with Schwab et al. study on patients hospitalised due to Covid-19, where the following changes in the oral mucosa were detected: dryness, erythema, atrophy, cracks/fissures, oropharyngeal secretions, petechiae, spontaneous bleeding, blood clots, traumatic ulcers and remains consistent to our observations [59]. The development of atrophic changes often occurs due to haematological and vitamin deficiencies, in immunological disorders (decreased immunity or exacerbation of autoimmune diseases) and in the course of opportunistic infections, such as candidiasis [60]. Angular cheilitis, found in more than half of the study group, is most often caused by a mixed streptococcal and fungal infection. Still, its occurrence is favoured by vitamin deficiencies, mainly B vitamins, iron and salivary disorders [60]. Oral candidiasis is a typical example of an opportunistic infection, developing in favourable conditions of immune deregulation, which makes the group of patients with Covid-19 particularly vulnerable to its development.

Summing up, oral mucosa status and the occurrence of symptoms on the mucosa correlated with the more severe course of the disease and the need for hospitalisation. Therefore, one must agree that dentists’ support could counteract side effects and thus prevent permanent changes in oral homeostasis, which is also crucial for their health in the future. Multidisciplinary support, including professional dental care, in the case of patients’ severe conditions, seems to be not only an empathetic or ethical but definitely a therapeutic indication [16, 20, 50–52].

Of the biochemical parameters, elevated D-dimer levels may be explained by comorbidity, dysfunction of endothelial cells, thrombin production, hypoxia and age-related long-term hospitalization of Covid-19 patients [61, 62]. D-dimer is usually monitored to avoid a thrombosis in the lung and mortality in Covid-19 [63]. The significant association to oral inflammation may confirm a possible role of oral infection, such as active caries and periodontitis [64–66]. Likewise, the found imbalance in other biochemical parameters, such us D-dimers, lymphocytes, neutrofile, IL-6, CRP may explain our clinical Beck’s score results.

Our results connected the poorer oral health related-condition with the need of non-invasive ventilation in COVID patients. The severe COVID-19 related to pneumonia and (as a result) respiratory failure were the most common cause of death among COVID-positive patients [67]. Severe COVID-associated pneumonia was caused not only by SARS-CoV-2, but also (even mainly) by acute immunological response for SARS-CoV-2 infection—this hyper reaction is diagnosed as a macrophage activation syndrome MAS or cytokine storm [68]. We can observe the elevating IL-6, TNF and IFNƳ serum levels in patients with severe COVID pneumonia, especially complicated by acute respiratory distress syndrome ARDS [68]. In acute inflammation, IL-6 induces a large number of acute phase proteins, e.g., CRP and serum amyloid A (SAA), so IL-6 takes a crucial role in acute inflammatory response [67].

A vast metanalysis (11 studies, 1302 Covid-19 patients) concluded that IL-6 serum level was 2.0 fold higher in patients with complications compared with those without complications [69]. The IL-6 level measured on admission to the hospital can be a prognostic factor of mortality and Intensive Care Unit ICU admission [70–72]. The IL-6 elevation can be used for early recognition of severe COVID-19 complications [73]. Among patients admitted to the ICU the IL-6 level positively correlated with organ failure severity, clinical worsening in future [74]. The IL-6 serum level can also be a predictor of in-hospital mortality in patients with severe COVID pneumonia admitted in the ICU [67, 75].

Knowledge of the role IL-6 in fatal consequences of COVID infection, enabled to use tocilizumab—the IL-6 receptors blocker. This drug is intended only for patients with respiratory failure and highly elevated IL-6 level (according to various sources: more than 75 and 100). The injection of Tocilizumab in those patients lowered the risk of the ICU admission, prolonged hospitalization and death [75, 76].

Whether periodontitis and carious lesions in teeth contributed to elevated IL-6 level need to be discussed. The process of periodontitis consists of direct damage caused by bacteria and immune processes as a well [77, 78]. The gingival fibroblasts contribute to pathogenesis by possessing a secretory phenotype characterized by an exuberant secretion of inflammatory mediators and cytokines [79], for example IL-6, IL-*β*, TNF and many others [78]. They usually mediate the physiological inflammatory process, but elevated chronically or inadequate may lead to tissue damage. There are many data, in which elevated levels of IL-6 can be observed in gingival crevicular fluid GCF samples collectedfrom periodontium [77–82]. There is also an association between some IL-6 gene polymorphism and aggressive periodontitis [83]. It was also proven, that the effective nonsurgical treatment of periodontitis leads to decrease of IL-6 on GCF [84]. There is a possibility to use the IL-6 level even to value a periimplantitis as bone resorbing factor [77, 78]. It has also be shown that elevated concentration of IL-6 not only in GCF was observed, but also in serum [83, 84]—as it was measured in COVID-19 positive patients admitted to the hospital.

**Fig. 2:**
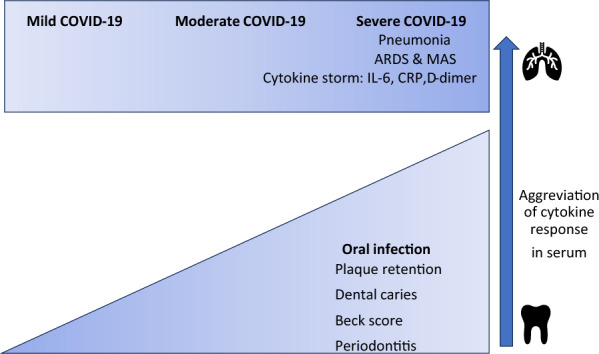
Mode of action showing cytokines and oral factors in COVID-19 severity. *ARDS* Macrophage Activation Syndrome-Like Disease, *MAS* macrophage activation syndrome

In our study, we proved that COVID-19 hospitalised patients with severe symptoms crossing with poor oral health-related conditions. In other studies there is a link between periodontitis and COVID severity patients with the poorer oral health had higher risk of assisted ventilation, ICU admission and death [42]. There is a hypothesis that the periodontal pockets can be reservoirs for SARS-CoV-2 [85] and periodontitis (through the synergistic activation of peripheral polymorphonuclear leukocytes to local and remote inflammatory triggers) can prime the immune system toward an exacerbated response for COVID-19 infection, and play a role in cytokine storm [85] (Fig. 2).

Some limitations of our observations should be pointed out. The patients were examined only once, so it was not possible to determine whether pathological changes in the oral cavity preceded Covid-19 infection or whether they developed during infection. Due to the coexistence of many systemic diseases in the examined persons, some of the oral lesions could have been caused by factors other than Covid-19 infection. Therefore, it is essential to consider whether these oral lesions are directly related to or secondary to SARS-CoV-2 infection. Longer follow-up periods may be suggested to evaluate the clinical approach to oral health among post-Covid-19 patients [86, 87].

Clinical evaluation was based on visual criteria during oral examination. Dental examinations did not include a dermatological evaluation of the facial skin. In assessing dental/bone damage, digital devices could be implemented, e.g., X-ray pictures, DIAGNOdent Pen, DIAGNOcam and CarieScan PRO. However, equipment use would be impossible due to sanitary regulations and patient isolation in the hospital. From a scientific perspective, the visual indexes make the results comparable to previously performed studies that focused on Covid-19 patients [6, 15, 37, 46]. However, there are still few published studies with the clinical evaluation of this group of critically ill patients.

One of the limitations of the study was the unknown educational level attained (primary and high school, higher education such as college/university) and employment status (employed, unemployed, student, retired). Due to the old age of the subjects, we suspect that most had retired and were not actively working.

Therefore, other sociodemographic confoundings could occur, like differences in socioeconomic status, income and family support. Social isolation and loneliness were negatively distinct during the lockdown periods, impacting emotional distress, sleeping, and nutritional habits [15, 16, 46]. During the pandemic time, a psychological aspect of dental anxiety before dental visits must be taken into account [15, 16, 46]. Any microbiological analysis from oral cavity which might indicate bacterial shifts was not performed. Nevertheless, one may argue that we examined a small cohort of hospitalised Covid-19 patients under intensive medical care. To our knowledge, this is the first clinical trial that compared such a group of Covid-19 patients in terms of egilibility in pulmonary symptoms, senior age, living area, and disease duration. Even if our results are not enough to prove oral conditions among the subjects, our limitations do not undermine the representativeness of the examined group.

## Conclusions

The negative influence of the severe Covid-19 course on oral health was manifested. Not without significance was their poor oral status before the viral infection, which intensified oral inflammation and hindered dental hygiene maintenance due to intensive general treatment protocols. Particurarly elevated IL-6, D-dimer, CRP levels in patients having oral inflammation and symptomatic SARS-CoV-2 may affect hospitalization outcomes. To inhibit extra- and intra-oral complications, a targeted intervention consisting of antibacterial, protective and moisturising oral mucosa is recommended. A point-of-care system together with the personalized oral examination could be a step forward in the management of Covid-19 patients under intensive hospital treatment due to pulmonary adverse outcomes.

### Supplementary Information

**Additional file 1: Table S1.** BRUSHED oral assessment model [30].

**Additional file 2: Table S2.** Beck's Oral Assessment Tool [31].

## Data Availability

The datasets used and/or analyzed during the current study are available from the corresponding author on reasonable request.

## Abbreviations

ALT: Alanine transferase
anti-HCV: Anti-hepatitis C antibodies
anti-HIV: Anti-human immunodeficiency virus antibodies
APTT: Activated partial thromboplastin time
ARDS: Acute respiratory distress syndrome
AST: Asparagine transferase
Beck score: Beck assessment tool to achieve individual indications for oromucosal care
BMI: Body mass index
BOP: Bleeding on probing
BRUSHED: Presence of bleeding, redness, ulceration, saliva, external factors and debris for noting model
CAL: Clinical attachment
CRP: C-reactive protein
COPD: Chronic obstructive pulmonary disease
D: Number of decayed teeth
DD: D-Dimer
DMFT: Decayed, missing, filled teeth index
F: Number of filled teeth
GCF: Gingival crevicular fluid
GGTP: Gamma glutamyl-transpeptidase
Hbs ag: Hepatitis B surface antigen
HRCT: High-resolution computed tomography
ICU: Intensive Care Unit
LDH: Lactate dehydrogenase
ICH: International Conference on Harmonization
IL-6: Interleukin-6
IL-*β*: Interleukin-*β*
INR: International normalised ratio
M: Number of missing teeth
n: Number of patients
MAS: Macrophage activation syndrome
OR: Odds ratio
PCT: Procalcitonin
PD: Probing depth
PCR: Plaque control record index
SAA: Serum amyloid A
TNF: Tumor necrosis factor
VAP: Ventilator pneumonia
WBC: White blood cells
WHO: World Health Organization
y: Years
