# Supplementary material for: Prevalence of oral complications in the course of severe SARS-CoV-2 infection under mechanical non-invasive ventilation
Source: Eur J Med Res. 2023 Aug 22;28:293. doi: 10.1186/s40001-023-01273-6 (PMC10463896; doi:10.1186/s40001-023-01273-6)
Supplement: Supplementary file 1 — Additional file 1: Table S1. BRUSHED oral assessment model [30]. [file 40001_2023_1273_MOESM1_ESM.docx]

Table S1. BRUSHED oral assessment model (Hayes and Jones 1995).

| **B** | BLEEDING?  (gums, mucosa, coagulation status?) |
| --- | --- |
| **R** | REDNESS?  (gum margins, tongue, antibiotic stomatitits?) |
| **U** | ULCERATION?  (size, shape, herpetic, infected?) |
| **S** | SALIVA?  (xerostomia, hypersalivation, characteristics?) |
| **H** | HALITOSIS?  (character, acidotic, infected?) |
| **E** | EXTERNAL FACTORS?  (angular chelitis, endotracheal tapes?) |
| **D** | DEBRIS?  (visible plaque, foreign particles?) |
