# Supplementary material for: Prevalence of oral complications in the course of severe SARS-CoV-2 infection under mechanical non-invasive ventilation
Source: Eur J Med Res. 2023 Aug 22;28:293. doi: 10.1186/s40001-023-01273-6 (PMC10463896; doi:10.1186/s40001-023-01273-6)
Supplement: Supplementary file 2 — Additional file 2: Table S2. Beck's Oral Assessment Tool [31]. [file 40001_2023_1273_MOESM2_ESM.docx]

Table S2. Beck's Oral Assessment Tool (Beck 1979)

| CATEGORIES | POINTS | | | |
| --- | --- | --- | --- | --- |
|  | 1 | 2 | 3 | 4 |
| Lips | smooth, moist  and not cracked | red, somewhat  dry | dry, swollen, slim  pocks | edematous,  inflamed pocks |
| Gums and mucosa | smooth, moist  and not cracked | faint, dry,  isolated lesions | swollen, red | inflamed, very  dry and  edematous |
| Tongue | smooth, moist  and not cracked | dry, visible  papilla | dry, swollen, red  with papilla  lesions | very dry,  edematous,  swollen lesions |
| Teeth | clean, no debris | small amount of  debris exists | moderate amount  of debris exists | full of debris |
| Saliva | light, succulent  and abundant | increasing on its  amount | insufficient and a  bit dense | very dense and  sticky |
| Total Points | 5 points  no dysfunctions  at least every 12  hours oral care | 6-10 points  slight Dysfunction  at least every 8-12 hours of oral care | 11-15 points  moderate amount of disfunction  at least every 8  hours of oral care | 16-20 points  serious amount  of disfunction  at least every 4  hours of oral care |

Explanations:

- 0–5 points: Do the oral assessment once a day. Perform the systematically prepared oral care protocol twice a day.
- 6–10 points: Do the oral assessment twice a day. Moisturise the lips and mucosa every 4 hours. Perform the systematically prepared oral care protocol at least twice a day.
- 11–15 points: Do the oral assessment every 8-12 hours. Perform the systematically prepared oral care protocol at least every 8 hours. Use a soft toothbrush. Moisturise the lips and mucosa every 2 hours.
- 16–20 points: Do the oral assessment and the oral care protocol every 4 hours. If you can't brush your teeth, use a wrapped-up gauze pad. Moisturise the lips and mucosa every 1-2 hours (Beck, 1979).
